# Notes and News

**Published:** 1895-12-14

**Authors:** 


### NOTES AND NEWS.

(Collected and Communicated.)

Small-pox is stated to be raging at Rio Janeiro, the mortality being so great as to cause almost a panic amongst the inhabitants.

The intended additions and improvements at the Fulham Infirmary will, besides increased accommodation for the nursing staff, consist of new kitchens and a new drainage scheme.

In a report presented to his Vestry by Dr. Waldo. on his recent action against the Salvation Army Shelter, he remarks that the weak point in the present position appears to lie in the fact that the sanitary officers have no power of entry at night for purposes of inspection; and he expresses the hope that as a result of the recent action it will sooner or later be found possible to bring all night refuges under the control of the Common Lodging House Acts.

Guy's Hospital is in want of money. There is a yearly deficiency of £20,000 which has to be made up somehow or other, and Guy's men have set about doing it in the right way—not by hope but by work. For years Guy's has been a great school, turning out men who now occupy responsible positions in every quarter of the globe, and it is to them that their old hospital and their old school must look for new strength. "The Guy's Hospital Gazette" has issued a "call to arms," an appeal to every Guy's man to do his best, in his own circle, to turn the stream of charity so far as he can influence it towards the coffers of his old school, and we cannot doubt that if the alumni of that great school will but put their shoulders to the wheel, and each do his best for the *alma mater* to whom they owe so much, Guy's Hospital will, after all its trials, be rich once more.

Cholera has reappeared in St. Petersburg, though the epidemic is not at present rapid in its growth In Egypt the disease is still confined to the districts of Diametta and Lake Menzaleh; the cases have not numbered 1,000, but the death-rate has been very large.

Mr. Sydney Holland's appeal for funds to erect isolation accommodation at the Poplar Hospital for Accidents, the urgent need of which was so strongly illustrated recently, is meeting with hearty response. The *Daily Chronicle* has opened its columns for subscriptions, and upwards of £2,400 has been already subscribed in addition to £2,000 for an Endowment Fund.

Of the £100,000 appealed for by St. Thomas's Hospital to enable the long-closed wards to be utilised, upwards of £26,000 has been subscribed. The governors have decided to throw open two wards containing sixty beds in January next, trusting to meeting with a continuance of support to enable them to be maintained.

Sir Henry Acland has been presented with a testimonial by the University of Oxford in recognition of his valuable services in the cause of medical science and hygiene during the forty years of his tenure of the office of Regius Professor of Medicine. The memorial takes the form of a bust to be placed in the museum and of £3,000 to be added to the funds of the Sarah Acland Home for Nurses, thus connecting him in these memorials with both science and charity.

The Italian Hospital Charity has recently been honoured by a special mark of the favour of Her Majesty the Queen of Italy, who has always taken much interest in its welfare, it having been founded by the munificence of Comm. G. B. Ortelli, and since managed by a committee of Italian and English gentlemen. The Queen's interest, real kindly sympathy, has been shown by the gift of a large and very beautiful portrait of herself, to which Her Majesty has added the following words in her own handwriting: "Possono gli Italiani racolti in questo luogo ritrovare sotto le benefiche ali della carità, la patria lontana nello spazio ma sempre presente nel desiderio del cuore.—Margherita."

The Christmas Appeal Supplement which we issue to-day is a reliable guide to all who desire to give to charities of proved utility. It is remarkable from the circumstance that the advertisements it contains constitute most interesting reading. Here each committee or secretary tells his own tale, and we hope that the charitable will study the supplement very carefully, and that any of them who wish for further bankers' forms will write to 428, Strand, for them. This supplement has its sad side, seeing that we have felt it necessary to exclude the advertisements of certain institutions where evidence of good management is lacking, a fact which should ensure a liberal response to the appeals of every charity which it contains. We hope, further, that the wealthy and other liberal givers will preserve our Christmas supplement, so that they may refer to it for information when they have money available for distribution at any time during the ensuing year.

